# Association Between COVID-19 and Self-Harm: Nationwide Retrospective Ecological Spatiotemporal Study in Metropolitan France

**DOI:** 10.2196/52759

**Published:** 2024-08-27

**Authors:** Maëlle Baillet, Marielle Wathelet, Antoine Lamer, Camille Frévent, Thomas Fovet, Fabien D'Hondt, Charles-Edouard Notredame, Guillaume Vaiva, Michael Génin

**Affiliations:** 1 Univ. Lille, CHU Lille, ULR 2694 - METRICS: Évaluation des technologies de santé et des pratiques médicales, Lille, France; 2 Univ. Lille, UFR 3S, Faculté Ingénierie et Management de la Santé, Lille, France; 3 Univ. Lille, INSERM, CHU Lille, U1172 - Lille Neuroscience & Cognition, Lille, France; 4 F2RSM Psy - Fédération régionale de recherche en psychiatrie et santé mentale Hauts-de-France, Saint-André-Lez-Lille, France; 5 Centre National de Ressources et de Résilience Lille-Paris, Lille, France

**Keywords:** self-harm, COVID-19, spatiotemporal analysis, ecological regression, data reuse

## Abstract

**Background:**

The COVID-19 pandemic has not been associated with increases in suicidal behavior at the national, regional, or county level. However, previous studies were not conducted on a finer scale or adjusted for ecological factors.

**Objective:**

Our objective was to assess the fine-scale spatiotemporal association between self-harm and COVID-19 hospitalizations, while considering ecological factors.

**Methods:**

Using the French national hospital discharge database, we extracted data on hospitalizations for self-harm of patients older than 10 years (from 2019 to 2021) or for COVID-19 (from 2020 to 2021) in metropolitan France. We first calculated monthly standardized incidence ratios (SIRs) for COVID-19 between March 2020 and December 2021, using a Besag, York, and Mollié spatiotemporal model. Next, we entered the SIRs into an ecological regression in order to test the association between hospital admissions for self-harm and those for COVID-19. Lastly, we adjusted for ecological variables with time lags of 0 to 6 months.

**Results:**

Compared with a smoothed SIR of ≤1, smoothed SIRs from 1 to 3, from 3 to 4, and greater than 4 for COVID-19 hospital admissions were associated with a subsequent increase in hospital admissions for self-harm, with a time lag of 2 to 4 months, 4 months, and 6 months, respectively.

**Conclusions:**

A high SIR for hospital admissions for COVID-19 was a risk factor for hospital admission for self-harm some months after the epidemic peaks. This finding emphasizes the importance of monitoring and seeking to prevent suicide attempts outside the epidemic peak periods.

## Introduction

The COVID-19 pandemic, which emerged in late 2019, swiftly evolved into a global crisis, prompting unprecedented measures such as lockdowns. Historically, epidemics and containment measures have been linked to adverse mental health outcomes [1]. As expected, early investigations in China revealed a surge in psychiatric disorders among COVID-19 survivors and in the general population [2].

The occurrence of mental health disorders, coupled with the impact of the pandemic on risk factors of suicide such as social isolation, precariousness, or reduced access to mental health care, has raised concerns about a potential rise in suicidal behaviors due to the COVID-19 pandemic [3,4]. The appearance of warning signs, such as increased consumption of psychotropic medications, has further heightened concerns on this subject [5,6]. Furthermore, cases of suicide deaths have already been observed during similar previous events such as during the Spanish flu in 1918‐1919 or during the severe acute respiratory syndrome outbreak in Hong Kong in 2003 [7].

Despite these different elements on which the concerns are based, the literature has not reported consistent evidence between the COVID-19 pandemic and suicidal behavior or self-harm. For instance, in France, Jollant et al [8] found a decrease in the number of hospital admissions for self-harm from January to August 2020 (compared for the same period in 2019) at the county level. Several studies of the whole of 2020 and even some of early 2021 have led to contradictory results. In some countries, there was no obvious increase in suicidal behavior and self-harm after the onset of the crisis, relative to previous years [9,10]. In others (eg, Japan), several studies found an initial decrease in suicide rates during the first months of the crisis and then an increase during the second half of 2020 (corresponding to the period between 2 lockdowns), before a return to the prepandemic level in early 2021 [11]. Finally, the most recent systematic review, including 34 studies, did not indicate a significant change in suicide rates during the COVID-19 pandemic. The pooled suicide rate in the studied period before the pandemic was 11.38/100,000 (95% CI 9.35‐13.42) and in the period during the pandemic was 10.65/100,000 (95% CI 8.61‐12.68) [12].

However, the majority of these studies were carried out at the national level and some focused on particular metropolitan regions [11] or a single region [10]. The use of these large-scale geospatial units potentiates the risk for ecological fallacy [13]. Spatiotemporal correlation analysis, conducted at a fine-scale and accounting for local socioeconomic factors and temporal trends in COVID-19 incidence, are lacking [14]. Indeed, given the heterogeneity across geographic regions, a nuanced examination of spatiotemporal suicide patterns, incorporating ecological variables such as social deprivation, urbanicity, and access to care, is imperative [15,16].

Therefore, our study aims to fill this gap by conducting a comprehensive ecological analysis to assess the fine-scale spatiotemporal association between hospital admissions for self-harm and COVID-19, while considering ecological factors such as social deprivation, living alone, accessibility to primary care, and urbanicity.

## Methods

### Study Setting and Data

This retrospective, observational study involved patients older than 10 years hospitalized in a medical, surgical, or obstetrics ward for self-harm from January 2019 to December 2021 or for COVID-19 from March 2020 to December 2021 in metropolitan France (mainland France and Corsica, an area covering a total of 543,940 km² and 57,249,208 inhabitants 10 years and older in 2018) [16]. A hybrid spatial unit, described in a previous work, was used to divide metropolitan France into 5535 spatial units [17]. The time unit was months.

Hospital stays were identified and extracted from the French Programme de médicalisation des systèmes d’information (PMSI; in English: National Hospital Discharge Database), which included details of all stays in the country’s public- and private-sector hospitals. In the PMSI, a unique national identifier for each patient enables all their hospital stays to be chained together. At the hospital stay level, the database contains information on the date of admission, the date of discharge, and sociodemographic variables (eg, the patient’s age, sex, and place of residence). For each stay, the principal diagnosis (ie, the reason for admission) and associated diagnoses (corresponding to the patient’s comorbidities) are also collected. The diagnoses are documented according to the French version of the *International Statistical Classification of Diseases, Tenth Revision* (*ICD-10*). Although only self-harm and COVID-19 requiring hospital admission can be identified, they correspond to what we can reasonably assume to be the most serious cases.

Regarding self-harm, all hospital stays that included *ICD-10* codes X60 through X84 for associated diagnoses in the 2019, 2020, or 2021 databases were included. We considered these associated diagnosis codes only because the PMSI does not allow them to be used for the principal diagnosis. Regarding COVID-19, all hospital stays including *ICD-10* codes U0710, U0711, U0714, and U0715 as the principal diagnosis in the 2020 or 2021 databases were included. Code U0712 was not included because it corresponds to asymptomatic COVID-19 and therefore was assumed not to be severe. Code U0713 was not included because it corresponds to examinations related to the COVID-19 outbreak and can only be used as an associated diagnosis.

The exclusion criteria were hospital stays (1) with an invalid anonymous code (making it impossible to chain together stays for a given patient), (2) with an admission date identical to the discharge date of a previous stay (corresponding to a hospital transfer, for example), (3) with a year of admission earlier than 2019 for self-harm and March 2020 for COVID-19 (since the stays are included in a database according to their year of discharge, and the codes for COVID-19 appeared in February 2020 only), (4) corresponding to recurrent stays in the same month for self-harm and at any time for COVID-19, (5) by patients younger than 10 years, and (6) with an invalid zip code or one corresponding to a French overseas region or dependency (making it impossible to identify the spatial unit).

Since we measured monthly incidences, we considered all recurrent stays for self-harm for the same patient during the same month as hospital readmissions after previous self-harm, and so this situation was not considered to be an incident case. In contrast, it was considered that all hospital readmissions of the same patient for COVID-19 were related to the first stay, since repeat infections were rare during this period [18]. All hospital readmissions of the same patient for COVID-19 were therefore not considered to be incident cases. For each stay ultimately selected, we extracted the patient’s sex, 5-year age group, and geographic code of the place of residence.

### Ecological Variables

The ecological variables considered were social deprivation, accessibility to a general practitioner (GP), urbanicity, and living alone. These ecological factors were chosen because they are known to be risk factors for suicidal behavior at the population level [19,20].

Social deprivation was estimated via the French Deprivation Index (FDep, issued by the French National Institute of Statistics and Economic Studies, INSEE), which considers the median income, the percentage of people with a high school leaving certificate, the percentage of manual and office workers, and the unemployment rate at the spatial unit level [19]. The FDep corresponds to the first component in a principal component analysis of these 4 variables: the higher the FDep, the greater the degree of social deprivation within the spatial unit.

Access to a GP was evaluated using the localized potential accessibility (LPA, an indicator developed by the French Directorate for Research, Studies, Evaluation and Statistics), calculated as the number of accessible consultations/visits per standardized population: the higher the LPA index, the greater the patients’ access to a GP [20].

Urbanicity was estimated by evaluating the percentage of artificialized surfaces in each spatial unit, as recorded in the Coordination of Information on the Environment Land Cover database [21]. We considered level 1, which corresponds to the main categories of land use identified on a global scale: urban features, industrial, commercial, and transport units, mines, dumps, construction sites, and artificial, nonagricultural green areas.

Living alone was defined as the ratio between the number of single-person households in the spatial unit and the total number of households in the spatial unit, according to the data reported by the INSEE [16].

All variables were obtained for 2018—the most recent year for which all were available. Since the spatial unit was the commune (municipality) in all cases, the data were aggregated at the spatial unit level by using the population-weighted average. This study’s reference population was that corresponding to people 10 years and older living in metropolitan France in 2018, according to data from the INSEE.

### Statistical Analyses

#### Overview

First, we calculated the monthly incidence rates of hospital admission for self-harm (from 2019 to 2021) and for COVID-19 (from March 2020 to 2021) in metropolitan France. The numerator corresponded to incident cases (ie, new cases of self-harm and COVID-19 in metropolitan France), and the denominator corresponded to the reference population. The incidence rates by 5-year age group and by sex were also calculated over the same period.

#### Spatiotemporal Modelization of Self-Harm and COVID-19 Incidences

##### Overview

In order to describe the spatiotemporal patterns of incidence of self-harm and COVID-19, we have specified a hierarchical Bayesian model that is commonly used in disease mapping [22,23]. Further, 2 separate models were considered for the incidence of self-harm and the incidence of COVID-19, respectively. In each model, the number of incident cases is modeled by a Poisson distribution in which the mean is a function of the expected number of incident cases, calculated by indirect age and gender standardization, using France as the reference population and several random effects capturing temporal and spatial patterns of incidence and their interactions. We fitted several spatiotemporal models with no covariates, including the spatial random effect and all combinations of temporal random effects and spatiotemporal interactions. The model with the best fit was chosen as the one minimizing the Watanabe-Akaike information criterion (WAIC). To describe the spatiotemporal patterns, we reported the smoothed spatiotemporal standardized incidence ratio (SIR) for each spatial unit and month over the period March 2020 to December 2021.

In terms of interpretation, a spatiotemporally smoothed SIR for hospital admissions for self-harm <1 or >1 corresponds to under- or overincidence, respectively, relative to the national monthly incidence in 2019. A smoothed SIR for COVID-19 hospital admissions <1 and >1 corresponds to an incidence below and above the national average, respectively, relative to the national average monthly incidence from March 2020 to December 2021. Mathematical formulations of the model, prior specifications, and model choice criterion are detailed in Multimedia Appendix 1 [22,24].

##### Ecological Regressions

To assess the association between hospital admissions for self-harm and those for COVID-19, we performed an ecological regression, that is, an extension of the previous modeling framework in which one or more covariates are included in the model as fixed effects. The effect of each covariate on hospital admission for self-harm was assessed by using the exponential of the coefficient associated with the covariate (the relative risk) and its Bayesian 95% credibility interval. The smoothed SIRs for hospital admissions for COVID-19 were transformed into classes: 0‐1, 1‐2, 2‐3, 3‐4, and >4. The reference class was class 0‐1, which corresponded to below-average incidence relative to the whole study area and study period. First, the SIRs for hospital admissions for self-harm were modeled by including the discretized smoothed SIRs of hospital admissions for COVID-19 calculated earlier as the only covariate. Given that COVID-19 might have had a time-lagged effect on hospital admissions for self-abuse, lags ranging from 0 to 6 months were tested. Second, the ecological variables were included in the ecological regression by using forward selection on the basis of the WAIC.

All models were estimated using the integrated nested Laplace approximation approach [25].

All analyses were carried out using RStudio (version 4.2.2; R Core Team) and the R-INLA package [25,26].

### Ethical Considerations

This observational study reused data from a national hospital discharge database. In France, this type of study does not require approval by an independent ethics committee. This study complied with the French government’s MR005 reference methodology, which governs access to PMSI data by health care institutions.

## Results

### Flowchart

**Figure 1.**
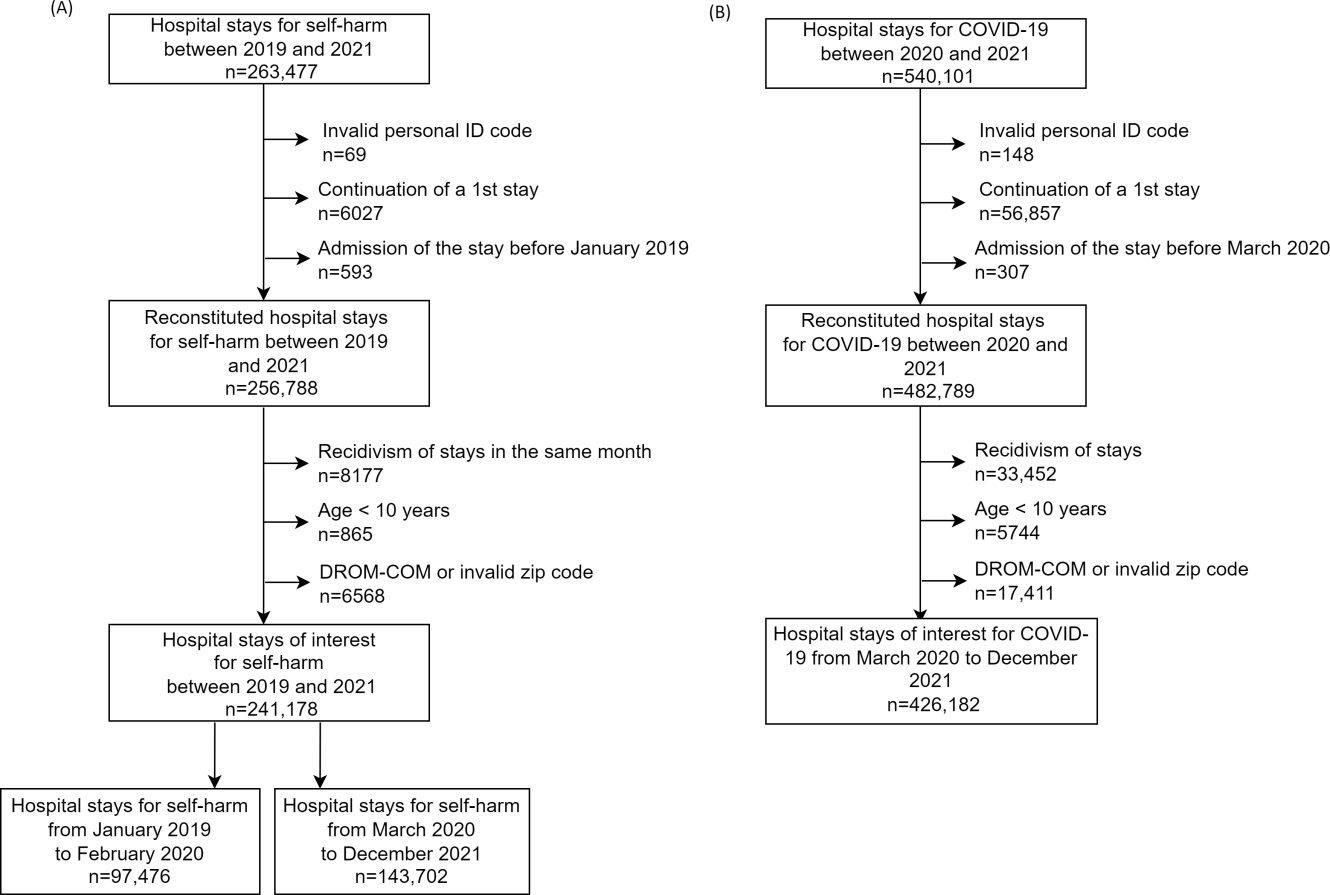
Flowchart hospital stays for (A) self-harm and (B) COVID-19. DROM-COM: Department Overseas Region and Overseas Collectivity.

A total of 241,178 stays for self-harm were extracted (97,476 between January 28, 2019, and February 2020, and 143,702 between March 2020 and December 2021; Figure 1). For COVID-19, 426,182 hospital stays were extracted.

### National Trends in the Monthly Incidence Rate

**Figure 2.**
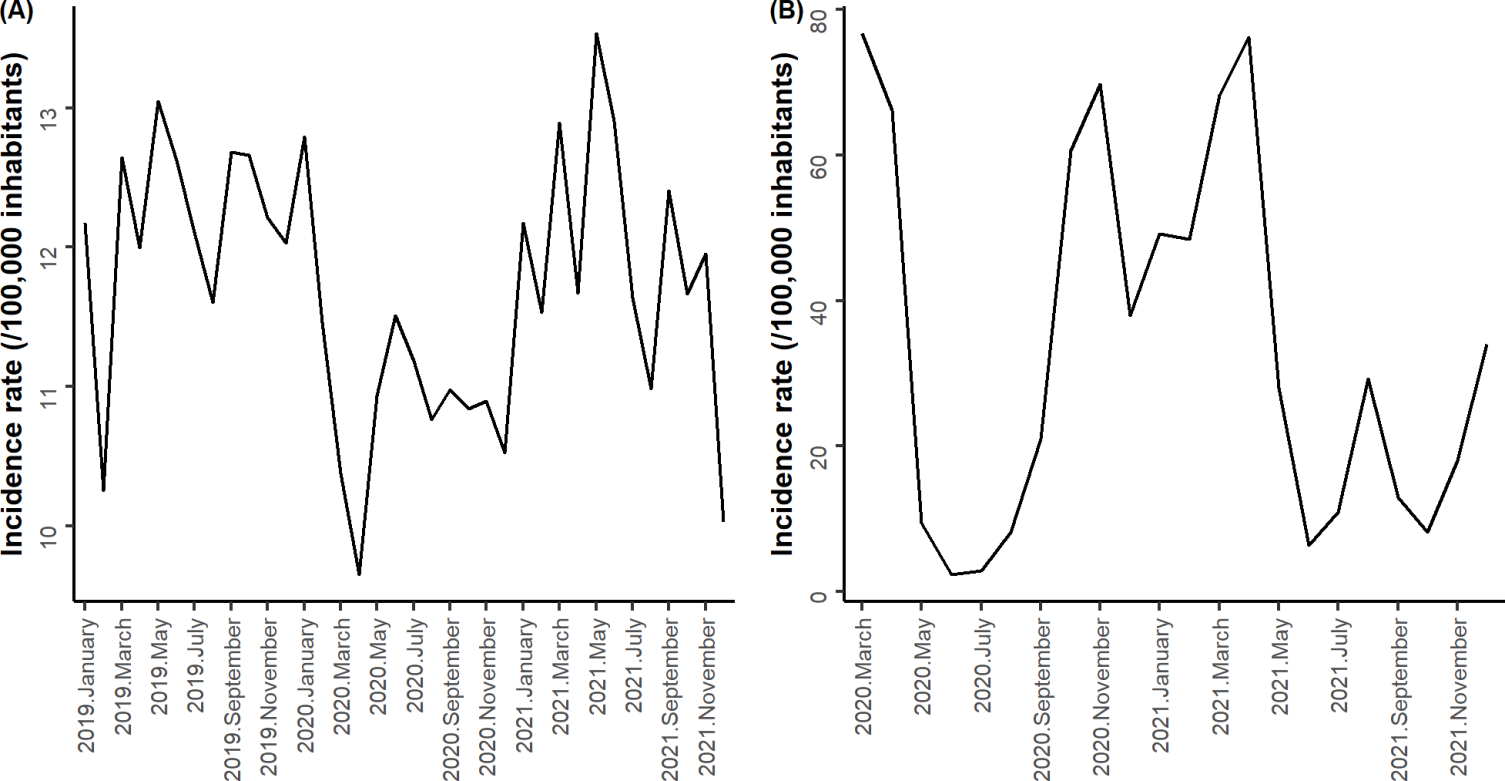
Changes in the monthly incidence rate of hospitalizations for self-harm from (A) January 2019 to December 2021 and for COVID-19 from (B) March 2020 to December 2021.

Concerning self-harm, a drop in the incidence rate was observed in February 2019, April 2020, and December 2021, with values of 10.25/100,000 inhabitants, 9.64/100,000 inhabitants, and 10.03/100,000 inhabitants, respectively (Figure 2). The incidence rate of self-harm in March to December 2020 was lower than in the same period in 2019 (9.64 to 11.50/100,000 inhabitants vs 11.60 to 13.05/100,000 inhabitants, respectively). The incidence rate of hospital admissions for self-harm peaked in May 2019 and May 2021, with values of 13.05/100,000 inhabitants and 13.53/100,000 inhabitants, respectively. Concerning the COVID-19 incidence rate, there were 3 main peaks in March 2020, November 2020, and April 2021, with values of 76.65/100,000 inhabitants, 69.74/100,000 population, and 76.19/100,000 inhabitants, respectively.

**Figure 3.**
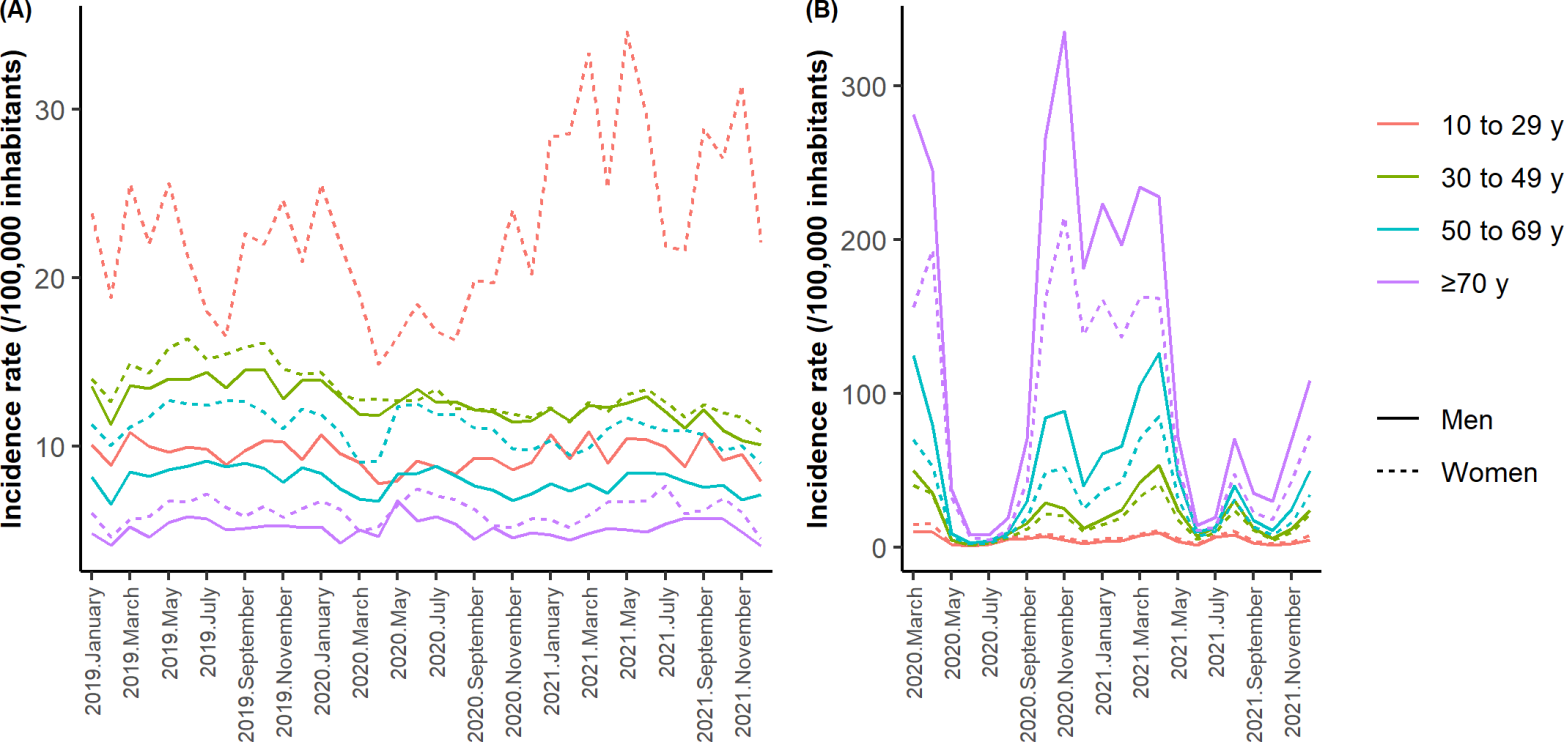
Changes in the monthly incidence rate of hospitalizations for self-harm from (A) January 2019 to December 2021 and COVID-19 from (B) March 2020 to December 2021 by age group and sex.

The incidence rate of hospital admissions for self-harm was higher for women than for men in all age groups (see Figure 3). The difference was greatest in the 10 to 29 years age group. A decrease in the incidence rate of hospital admissions for self-harm in all groups was observed to varying degrees in March and April 2020. There was an increase in the incidence rate of hospital admissions for self-harm in 2021 among young women aged 10 to 29 years but not in the other age groups of women or among men. In the group of women aged 10 to 29 years, the incidence rate also decreased in the months of July and August in all years. Regarding COVID-19, the incidence rate of hospital admissions was higher in men in all age groups. This incidence rate also increased with age, and the difference between men and women was greatest during periods with the high incidence rates.

### Spatiotemporal Models

Based on the selected models, the changes over time in the SIRs for self-harm and COVID-19 are presented in Multimedia Appendix 2, which also shows the timeline of the various health measures. The SIR for hospital admissions for self-harm appeared to be roughly stable at the spatial level over time. In contrast, there was more spatial and temporal variation in SIR for COVID-19 hospital admissions. The increase in the SIRs for COVID-19 hospital admissions coincided with the implementation of health measures, such as confinement. Conversely, the decrease in the SIRs coincided with the end of these measures.

### Assessment of the Fine-Scale Spatiotemporal Association Between Hospital Admissions for Self-Harm and Hospital Admissions for COVID-19

**Figure 4.**
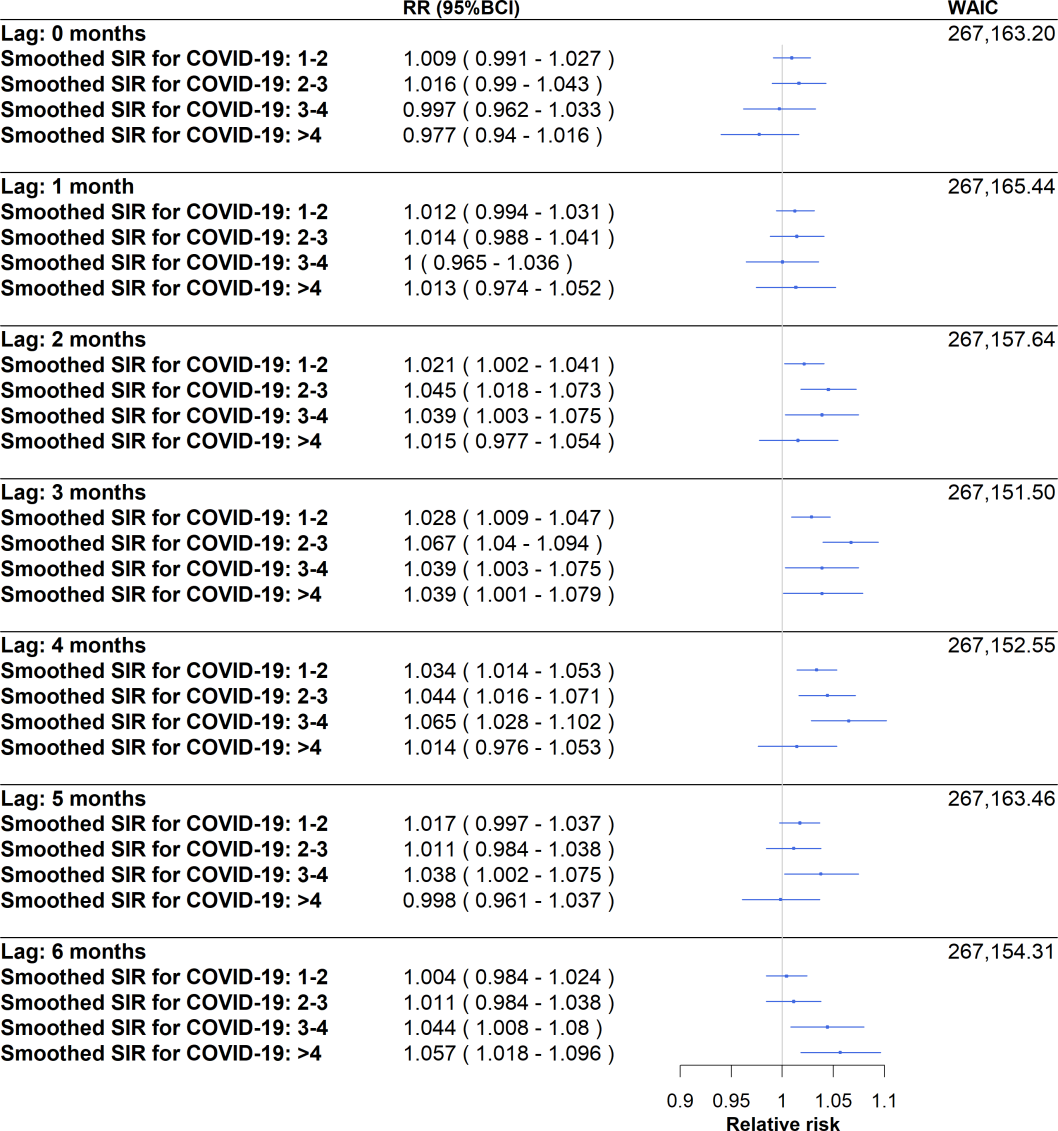
Associations between hospitalization SIR for COVID-19 and hospitalizations for self-harm with different lags between the 2 types of hospitalizations (0 to 6 months). For example, a 3-month lag is the SIR of COVID-19 hospitalizations at time T to assess the incidence rates of self-harm hospitalizations at T + 3 months. BCI: Bayesian credibility intervals; RR: relative risk; SIR: standardized incidence ratio; WAIC: Watanabe-Akaike information criterion.

Compared with the reference class (a SIR lower than or equal to 1, ie, spatiotemporal units with a below-average incidence rate for hospital admission for COVID-19 over this study’s period), several smoothed COVID-19 SIR classes greater than 1 had a statistically significant association with hospital admissions for self-harm over this study’s period (March 2020 to December 2021; Figure 4). At 2 months, a statistically significant effect was present in SIR classes 1‐2 (relative risk [RR] 1.021, 95% Bayesian credibility interval [BCI] 1.002‐1.041), 2‐3 (RR 1.045, 95% BCI 1.018‐1.073), and 3‐4 (RR 1.039, 95% BCI 1.003‐1.075). At 3 months, a statistically significant effect was present in SIR classes 1‐2 (RR 1.028, 95% BCI 1.009‐1.047), 2‐3 (RR 1.067, 95% BCI 1.040‐1.094), 3‐4 (RR 1.039, 95% BCI 1.003‐1.075), and >4 (RR 1.039, 95% BCI 1.001‐1.079). At 4 months, a statistically significant effect was present in SIR classes 1‐2 (RR 1.034, 95% BCI 1.014‐1.053), 2‐3 (RR 1.044, 95% BCI 1.016‐1.071), and 3‐4 (RR 1.065, 95% BCI 1.028‐1.102). At 5 months, a statistically significant effect was present for class 3‐4 (RR 1.038, 95% BCI 1.002‐1.075). Lastly, at 6 months, a statistically significant effect was present for classes 3‐4 (RR 1.044, 95% BCI 1.008‐1.080) and >4 (RR 1.057, 95% BCI 1.018‐1.096). No statistically significant effects were present at 0 or 1 months.

### Ecological Factors Associated With Hospital Admission for Self-Harm Incidence

**Figure 5.**
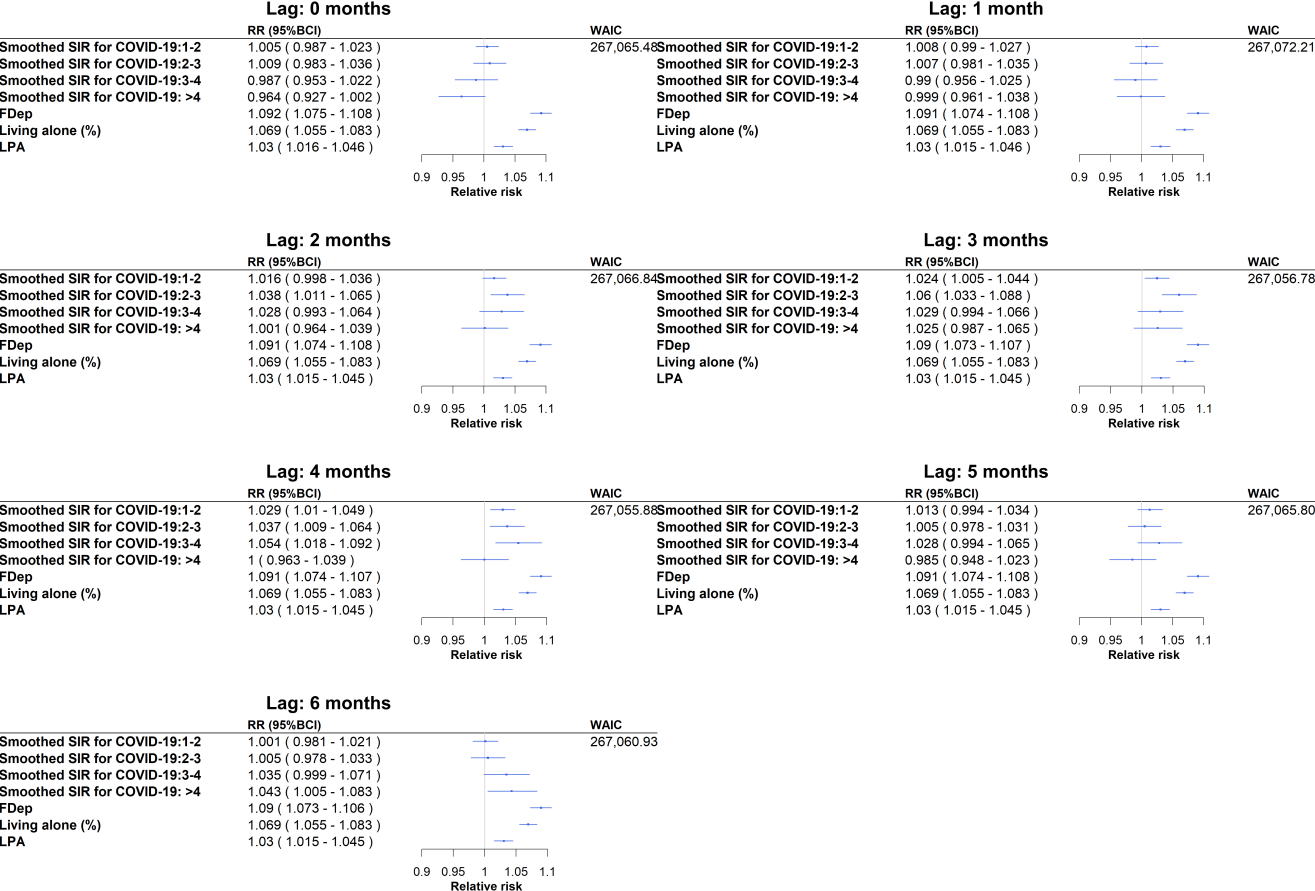
Ecological regression assessing the association between smoothed COVID-19 SIRs, ecological variables, and hospitalizations for self-harm with different lags (0 to 6 months). For example, a 3-month lag is the SIR of COVID-19 hospitalizations at time T to assess the SIRs of self-harm hospitalizations at T + 3 months. BCI: Bayesian credibility interval; FDep: French Deprivation Index; LPA: localized potential accessibility; RR: relative risk; SIR: standardized incidence ratio; WAIC: Watanabe-Akaike information criterion.

In ecological regressions (Figure 5), a high FDep (ie, high social deprivation), a high proportion of living alone, and a high LPA were identified as risk factors for the incidence rate of hospital admission for self-harm, whatever the time lag; for example, the relative risks were 1.091 (95% BCI 1.074‐1.107), 1.069 (95% BCI 1.055‐1.083), and 1.030 (95% BCI 1.015‐1.045), respectively, with a 4-month lag (the model with the lowest WAIC: 267,055.88). For the smoothed COVID-19 SIR, a statistically significant effect persisted in SIR class 2‐3 at 2 months (RR 1.038, 95% BCI 1.011‐1.065). At 3 months, a statistically significant effect persisted in SIR classes 1‐2 (RR 1.024, 95% BCI 1.005‐1.044) and 2‐3 (RR 1.060, 95% BCI 1.033‐1.088). At 4 months, a statistically significant effect persisted in SIR classes 1‐2 (RR 1.029, 95% BCI 1.010‐1.049), 2‐3 (RR 1.037, 95% BCI 1.009‐1.064), and 3‐4 (RR 1.054, 95% BCI 1.018‐1.092). No statistically significant effect persisted at 5 months. At 6 months, a statistically significant effect persisted in SIR class >4 (RR 1.043, 95% BCI 1.005‐1.083).

## Discussion

### Principal Results

In this study of data from metropolitan France, we studied the national monthly incidence rate of hospital admissions for self-harm from January 2019 to December 2021 and the rate for COVID-19 from March 2020 to December 2021. Regarding hospital admissions for self-harm, we observed a decrease in incidence rates from March 2020 to December 2020 (relative to the same period in 2019). In general, the incidence rates for hospital admissions for self-harm were higher among women. The effect was especially marked in the aged 10 to 29 years group and less marked in the aged 50 to 69 years group. The incidence rates were higher in women aged 10 to 29 years in all periods of interest. Moreover, this was the only class with a high incidence rate at the end of 2020, compared with the same period in 2019. Our spatiotemporal analyses of hospital admissions for self-harm showed that there were no clear spatial variations over time in the SIR. For COVID-19 hospital admissions, the SIR showed spatial and temporal variations consistent with the implementation of health measures. Social deprivation, living alone, and GP accessibility prior to the pandemic were risk factors for hospital admissions for self-harm. After adjustment for these ecological variables, the relationship between hospital admissions for self-harm and those for COVID-19 was not statistically significant when contemporaneous variations or a 1-month lag were modeled. In contrast, hospital admissions for COVID-19 were associated with an increase in hospital admissions for self-harm when a 2- to 6-month lag was modeled. It appeared that the time lag between hospital admissions for COVID-19 and those for self-harm was dependent on the SIR for COVID-19. Thus, a COVID-19 incidence 1 to 3 times the average over this study’s period and over the area (ie, a SIR from 1 to 3), 3 to 4 times the average (SIR from 3 to 4), and at 4 times or more of the average (SIR >4) were risk factors for self-harm hospital admission 2 to 4 months later, 4 months later, and 6 months later, respectively, compared with the reference below-average incidence rate (SIR ≤1).

### Comparison With Prior Work

Concerning the change in the incidence rate of hospital admissions for self-harm in metropolitan France between 2019 and 2020, the decrease observed in our study is consistent with previous studies in the field [8-10,27]. The predominance of hospital admissions for self-harm in women had already been observed in France by Chan-Chee [28] between 2008 and 2017. Moreover, this incidence rate predominated in young women aged 15 to 19 years in a manner consistent with the results of our study in which we find a predominance of the incidence rate in young women aged 10 to 29 years. Regarding the increase in the incidence rate of hospital admissions for self-harm for young women aged 10 to 29 years from the end of 2020, different studies have also found this effect in young women aged 10 to 18 years [9,29]. Multimedia Appendix 3 shows in more detail the change in the incidence rate among young people aged 10 to 29 years in our study and shows that this increase was also predominant among young women aged 15 to 19 and 10 to 14 years.

To our knowledge, the incidence rate of hospital admissions for self-harm in France had not yet been studied at such a fine spatiotemporal scale. In 2018, Chan-Chee [28] studied hospital admissions for self-harm in short-term care facilities between 2008 and 2017 at a regional and annual scale. In our study, the spatiotemporal distribution of hospital admissions for self-harm was spatially disparate but appeared to be rather stable at the temporal scale with a predominance of hospital admissions for self-harm, particularly in the North of France and in Bretagne (western tip of France). These results are consistent with the results published by Chan-Chee [28]. Concerning the spatiotemporal evolution of hospital admissions for COVID-19, the spatiotemporal distribution was more disparate. However, it was consistent with the chronology of the health measures implemented by the government.

Social deprivation, living alone, and accessibility to a GP in 2018 were risk factors of hospital admission for self-harm. Social deprivation [11] and social isolation [1,30] were already known to be risk factors for suicidal behaviors in the pre–COVID-19 period. Their effect therefore appeared to persist during the pandemic. GPs have an important role in suicide prevention [31]. However, the effect of GP accessibility on suicidal behaviors has not—to the best of our knowledge—been studied previously. Surprisingly, we found that access to a GP was associated with an increased risk of self-harm; this might be because the majority of suicide attempts involve voluntary drug self-administration [32]. Having access to a GP would give access to prescription medications [33]. This association might also be biased by confounding factors, since GP accessibility is usually higher in an urban environment. We did not find that urbanicity was a risk factor for hospital admission for self-harm—possibly because of its collinearity with access to a GP [15].

In this study, we assessed the fine-scale spatiotemporal association between hospital admissions for self-harm and COVID-19 and then adjusted the results for ecological variables known to be risk factors. The reference class was below-average hospital admission for COVID-19 over this study’s period and this study’s area (SIR ≤1). On average, a SIR for hospital admission for COVID-19 from 1 to 3 was a risk factor and ranged from 2.4% to 6% versus the reference class for hospital admissions for self-harm, with a lag of 2 to 4 months. A COVID-19 SIR from 3 to 4 was a risk factor (5.4%) versus the reference class for hospital admissions for self-harm, with a lag of 4 months. Lastly, a COVID-19 SIR greater than 4 was a risk factor (4.3%) versus the reference class for hospital admissions for self-harm, with a lag of 6 months. Hence, the higher the COVID-19 SIR, the longer the time lag to hospital admissions for self-harm. To the best of our knowledge, our study is the first to have looked at the average association between the SIRs for COVID-19 hospital admissions and self-harm hospital admissions. Although many studies have found a decrease in self-harm in 2020‐2021 versus 2019 [8-10,27], we evidenced a positive fine-scale association between hospital admissions for COVID-19 and those for self-harm over the 2020‐2021 period, with a lag of several months. One can hypothesize that COVID-19 impacted the occurrence of self-harm with a time lag, due to the above-mentioned direct and indirect consequences of the pandemic—notably an increase in mental health disorders in COVID-19 survivors and in the general population. Alternatively, one can hypothesize that COVID-19 did not have an impact on suicide attempts but that it reduced access to health care (due to lockdowns or fear of being contaminated in hospital). In that case, the delayed appearance of admission for self-harm would simply reflect the return to a normal situation, that is, the lifting of confinement and restrictions, the decongestion of care services, and hospital admission for a suicide attempt rather than staying at home.

### Implications and Perspectives

The findings of our study carry significant implications for public health policy and suicide prevention strategies in the context of a pandemic. The key implications of our study is the need for dynamic and adaptive suicide prevention strategies. Indeed, despite the overall decrease in hospital admissions for self-harm observed nationwide in 2020 compared to 2019, we found that hospital admissions for COVID-19 hotspots were associated with a delayed increase in hospital admissions for self-harm, with a lag of 2 to 6 months. Furthermore, our findings highlight the importance of addressing underlying social determinants of mental health, such as social deprivation and social isolation, which were identified as risk factors for hospital admissions for self-harm. Overall, this underscores the importance of proactive surveillance and targeted interventions to mitigate the impact of epidemic peaks on mental health outcomes, particularly suicidal behaviors. This may involve deploying resources and support services to at-risk areas, investing community-based interventions that promote social connectedness and provide support to vulnerable populations [34]. In addition to large-scale interventions, public policies must thus be implemented in the territories, consistent with local specificities, in order to reduce both suicidal behaviors and geographic disparities [35]. Fine-scale spatial analysis are thus necessary to identify at-risk areas and target prevention efforts, especially in health crisis contexts where resources are likely to be limited.

In addition to targeted interventions, there is a critical need for ongoing research to deepen our understanding of the complex relationship between the COVID-19 pandemic and mental health outcomes. Future studies should explore the underlying mechanisms driving the observed association between COVID-19 incidence and subsequent self-harm admissions, including the role of psychological distress, access to mental health services, and stigma surrounding help-seeking behaviors. Moreover, given the observed increase in hospital admissions for self-harm among adolescent girls in 2021, there is a pressing need for research focused on understanding and addressing the unique mental health challenges faced by this demographic group [36]. Spatial-temporal studies offer an analytical framework to better understand the geographical and temporal variations in suicidal behaviors, including the observed increase in suicide among young girls during the COVID-19 crisis. These studies can help identify geographic clusters where suicide rates among young girls have disproportionately increased during the COVID-19 crisis, provide an analysis of temporal trends to assess the long-term impact of the pandemic, and explore the social determinants of suicidal behaviors among girls. This may include factors such as social isolation, family stress, economic hardships, and access to mental health services. Coupled with qualitative research, this could help in understanding the phenomenon and provide tailored solutions to address it.

### Strengths

Our study had several strengths. It is the first to have evaluated the distribution of hospital admissions for self-harm and for COVID-19 in France on a fine spatiotemporal scale, and different time lags in the average association between these 2 variables. The spatiotemporal distribution of hospital admissions for self-harm and for COVID-19 were analyzed using a robust statistical method, with adjustment for spatial and temporal autocorrelations. In the Bayesian hierarchical Poisson model spatiotemporal model for hospital admissions for self-harm, the reference was the number of cases expected with the same incidence as before the pandemic in 2019. The fine-scale analysis enabled us to reduce sources of ecological biases. Lastly, we adjusted the models for ecological variables known to be risk factors for hospital admission for self-harm.

### Limitations

Our study also had some limitations. The COVID-19 incidence and the ecological variables were assessed on the territory scale, and so the associations highlighted in this study cannot therefore be considered at the individual level. The PMSI database does not contain data on patients who are self-harming but are not hospitalized. Hence, self-harm may have been underestimated, particularly in the context of COVID-19. Indeed, one can hypothesize that the patients who would normally have been hospitalized were not hospitalized (due to a lack of hospital resources) or that patients feared contamination and were less likely to attend the emergency department. Thus, we assumed that patients hospitalized for self-harm or for COVID-19 presented the most severe forms of these conditions. Although, the codes X60 to X84 are regularly used in the literature to identify self-harm, they can be only associated diagnoses and so might have been less well coded than the other types of diagnoses; this might have led to underestimation. Another limitation related to the PMSI database concerned the difficulty in distinguishing successive hospital admissions for the same self-harm incident from hospital admission for a new self-harm incident. We decided to select only the first self-harm incident in the monthly at the individual level but we could not be certain that this was a new self-harm incident and not a follow-up admission for a previous self-harm incident. These PMSI-related limitations cannot be circumvented, and there is no national-scale cohort that would allow us to take all the self-harm incidents into account. We therefore assume that the results of this study provide an initial estimate. Last, the data on the ecological variables came from 2018, that is, before the pandemic period; however, more recent data were not available. If the health crisis had had an impact on these variables, it would not have been taken into account in this study. Social isolation is a significant risk factor for suicidal behavior. Social isolation cannot be reduced to living alone and involves other components such as frequency of contact with family or friends, participation in group activities, etc. However, due to the ecological variables available to us, we were only able to use the fact of living alone to approach social isolation.

### Conclusions

Our results showed that despite the nationwide decrease in hospital admissions for self-harm in 2020 (compared with 2019), the frequency of hospital admission for COVID-19 in the space-time unit was a risk factor for subsequent hospital admission for self-harm. The time lag between the 2 types of admission ranged from 2 to 6 months; the higher the SIR for hospital admission for COVID-19, the greater the time lag to a hospital admission for self-harm. Our results show that it is important to monitor and seek to prevent suicide attempts away from epidemic peaks. Future research should focus on the spatiotemporal changes in self-harm (compared with 2019) and particularly on the situation among adolescent girls, given the observed increase in hospital admissions for self-harm in this group in 2021.

## Supplementary material

10.2196/52759 Multimedia Appendix 1 Spatiotemporal models and ecological regression.

10.2196/52759 Multimedia Appendix 2 Timeline of the distribution of standardized incidence ratios for self-harm and COVID-19 with a timeline representing the implementation of health measures by month.

10.2196/52759 Multimedia Appendix 3 Changes in the incidence rate of hospital admissions for self-harm from January 2019 to December 2021 in people aged between 10 and 29 years.

## Abbreviations

BCI: Bayesian credibility interval
FDep: French Deprivation Index
GP: general practitioner
*ICD-10*: *International Statistical Classification of Diseases, Tenth Revision*
INSEE: French National Institute of Statistics and Economic Studies
LPA: localized potential accessibility
PMSI: Programme de médicalisation des systèmes d'information (English: French National Hospital Discharge Database)
RR: relative risk
SIR: standardized incidence ratio
WAIC: Watanabe-Akaike information criterion

## References

[1] Brooks SK, Webster RK, Smith LE (2020). The psychological impact of quarantine and how to reduce it: rapid review of the evidence. Lancet.

[2] Xiong J, Lipsitz O, Nasri F (2020). Impact of COVID-19 pandemic on mental health in the general population: a systematic review. J Affect Disord.

[3] Moitra M, Santomauro D, Degenhardt L (2021). Estimating the risk of suicide associated with mental disorders: a systematic review and meta-regression analysis. J Psychiatr Res.

[4] Gunnell D, Appleby L, Arensman E (2020). Suicide risk and prevention during the COVID-19 pandemic. Lancet Psychiatry.

[5] Levaillant M, Wathelet M, Lamer A, Riquin E, Gohier B, Hamel-Broza JF (2023). Impact of COVID-19 pandemic and lockdowns on the consumption of anxiolytics, hypnotics and antidepressants according to age groups: a French nationwide study. Psychol Med.

[6] Reger MA, Stanley IH, Joiner TE (2020). Suicide mortality and coronavirus disease 2019-a perfect storm?. JAMA Psychiatry.

[7] Yip PSF, Cheung YT, Chau PH, Law YW (2010). The impact of epidemic outbreak: the case of severe acute respiratory syndrome (SARS) and suicide among older adults in Hong Kong. Crisis.

[8] Jollant F, Roussot A, Corruble E (2021). Hospitalization for self-harm during the early months of the COVID-19 pandemic in France: a nationwide retrospective observational cohort study. Lancet Reg Health Eur.

[9] Steeg S, Bojanić L, Tilston G (2021). Temporal trends in primary care-recorded self-harm during and beyond the first year of the COVID-19 pandemic: time series analysis of electronic healthcare records for 2.8 million patients in the Greater Manchester Care Record. EClinMed.

[10] Matsumoto R, Motomura E, Fukuyama K, Shiroyama T, Okada M (2021). Determining what changed Japanese suicide mortality in 2020 using governmental database. J Clin Med.

[11] Rehkopf DH, Buka SL (2006). The association between suicide and the socio-economic characteristics of geographical areas: a systematic review. Psychol Med.

[12] da Cunha Varella AP, Griffin E, Khashan A, Kabir Z (2024). Suicide rates before and during the COVID-19 pandemic: a systematic review and meta-analysis. Soc Psychiatry Psychiatr Epidemiol.

[13] Morgenstern H (1982). Uses of ecologic analysis in epidemiologic research. Am J Public Health.

[14] Rezaeian M, Dunn G, St Leger S, Appleby L (2006). Ecological association between suicide rates and indices of deprivation in the North West region of England: the importance of the size of the administrative unit. J Epidemiol Community Health.

[15] Satherley RM, Hazell CM, Jones CJ, Hanna P (2022). A systematic review of the effects of urban living on suicidality and self-harm in the UK and Ireland. J Urban Health.

[16] National Institute of Statistics and Economic Studies.

[17] Ghenassia A, Beuscart JB, Ficheur G (2017). A generic method for improving the spatial interoperability of medical and ecological databases. Int J Health Geogr.

[18] Sotoodeh Ghorbani S, Taherpour N, Bayat S, Ghajari H, Mohseni P, Hashemi Nazari SS (2022). Epidemiologic characteristics of cases with reinfection, recurrence, and hospital readmission due to COVID-19: a systematic review and meta-analysis. J Med Virol.

[19] Pornet C, Delpierre C, Dejardin O (2012). Construction of an adaptable European transnational ecological deprivation index: the French version. J Epidemiol Community Health.

[20] Barlet M, Coldefy M, Collin C, Lucas-Gabrielli V (2012). L’accessibilité aux médecins généralistes libéraux: plus faible en milieu rural. Pour.

[21] (2018). CORINE land cover. Données et études statistiques.

[22] Besag J, York J, Mollié A (1991). Bayesian image restoration, with two applications in spatial statistics. Ann Inst Stat Math.

[23] Knorr-Held L, Best NG (2001). A shared component model for detecting joint and selective clustering of two diseases. J R Stat Soc Ser A Stat Soc.

[24] Knorr-Held L (2000). Bayesian modelling of inseparable space-time variation in disease risk. Stat Med.

[25] Rue H, Martino S, Chopin N (2009). Approximate Bayesian inference for latent Gaussian models by using integrated nested Laplace approximations. J R Stat Soc Series B.

[26] The R Project for Statistical Computing.

[27] John A, Eyles E, Webb RT (2021). The impact of the COVID-19 pandemic on self-harm and suicidal behaviour: update of living systematic review. F1000Res.

[28] Chan-Chee C (2019). Les hospitalisations pour tentative de suicide dans les établissements de soins de courte durée: évolution entre 2008 et 2017 [Article in French]. Bull épidém hebd.

[29] Yard E, Radhakrishnan L, Ballesteros MF (2021). Emergency department visits for suspected suicide attempts among persons aged 12-25 years before and during the COVID-19 pandemic - United States. MMWR Morb Mortal Wkly Rep.

[30] McClelland H, Evans JJ, Nowland R, Ferguson E, O’Connor RC (2020). Loneliness as a predictor of suicidal ideation and behaviour: a systematic review and meta-analysis of prospective studies. J Affect Disord.

[31] Mann JJ, Apter A, Bertolote J (2005). Suicide prevention strategies: a systematic review. JAMA.

[32] Fossi LD, Debien C, Demarty AL, Vaiva G, Messiah A (2021). Suicide reattempt in a population-wide brief contact intervention to prevent suicide attempts: the VigilanS program, France. Eur Psychiatry.

[33] Graetz N, Preston SH, Peele M, Elo IT (2020). Ecological factors associated with suicide mortality among non-hispanic whites. BMC Public Health.

[34] Alegría M, NeMoyer A, Falgàs Bagué I, Wang Y, Alvarez K (2018). Social determinants of mental health: where we are and where we need to go. Curr Psychiatry Rep.

[35] Kassem AM, Carter KK, Johnson CJ, Hahn CG (2019). Spatial clustering of suicide and associated community characteristics, Idaho, 2010-2014. Prev Chronic Dis.

[36] Wong BHC, Cross S, Zavaleta-Ramírez P (2023). Self-harm in children and adolescents who presented at emergency units during the COVID-19 pandemic: an international retrospective cohort study. J Am Acad Child Adolesc Psychiatry.

