# Supplementary material for: Association Between COVID-19 and Self-Harm: Nationwide Retrospective Ecological Spatiotemporal Study in Metropolitan France
Source: JMIR Public Health Surveill. 2024 Aug 27;10:e52759. doi: 10.2196/52759 (PMC11370185; doi:10.2196/52759)
Supplement: Multimedia Appendix 1 [file publichealth-v10-e52759-s001.pdf]

Multimedia appendix 1

1 Spatio-temporal models and ecological regressions

1.1 Spatio-temporal models

Let denote by y_{it} and E_{it} the observed number and age-and -gender standardized expected number of either hospital admissions for self-harm or COVID-19 incident cases for the ith spatial unit and t-th month t, respectively. The expected number of hospital admissions for COVID-19 was calculated as follows:

$$E_{it} = \sum_{k=1}^{K} n_{kit} p_k,$$

where n_{kit} is the population at risk in spatial unit i in month t and age and sex stratum k, and p_k corresponds to the incidence rate of COVID-19 in stratum k.

A more novel approach was used to calculate the expected number of hospital admissions for self-harm. In fact, the incidence of hospital admissions for self-harm before the pandemic was relevant for inclusion in the model because the association between hospital admissions for self-harm and COVID-19 might change over time. This information was included in the calculation of the expected number of cases, as follows:

$$E_{it} = \sum_{k=1}^{K} n_{kit} p_{kt}^{2019},$$

where p_{kt}^{2019} is the incidence rate of hospital admissions for self-harm in month t in 2019 in stratum k in the study area. This approach differs from the classical approach but has the advantage of using the 2019 monthly incidence rates as a reference.

The general formulation of the model is the following:

$$\begin{aligned}
y_{it} &\sim \text{Poisson}(\theta_{it} E_{it}) \\
\log(\theta_{it}) &= \beta_0 + u_i + v_i + \xi_t + \gamma_t + \delta_{it} \\
u_i | u_{-i} &\sim \text{Normal}\left(\frac{\sum_j w_{ij} u_j}{\sum_j w_{ij}}, \frac{\sigma_u^2}{\sum_j w_{ij}}\right) \text{ (ICAR)} \\
v_i &\sim \text{Normal}(0, \sigma_v^2) \\
\xi_t &\sim \text{Normal}(0, \sigma_\xi^2) \\
\gamma_t &\sim \text{RW}n(\sigma_\gamma^2) \\
\delta_{it} &\sim \text{Normal}(0, \sigma_\delta^2)
\end{aligned}$$

where θ_{it} is the relative risk of either hospital admissions for self-harm or COVID-19 incidence in the i-th spatial unit and t-th time, and β_0 is an intercept (overall risk). u_i is a spatially structured random effect modeled by an intrinsic conditional autoregressive model (ICAR) in which the weights w_{ij} of adjacent spatial units are equal to 1 and all others are equal to 0, of variance σ_u^2. v_i is an unstructured spatially random effect of variance σ_v^2. ξ_t is unstructured temporal random effet (i.i.d.) of variance σ_ξ^2 and γ_t is temporally structured random effect defined as a random walk of order n (RWn), $n = 1$ or 2, of variance σ_γ^2. δ_{it} corresponds to the type I interaction of both temporal and spatial components, referring to unstructured overdispersion in time and space, and modeled as an i.i.d. random effect of variance σ_δ^2 [Besag et al., 1991, Knorr-Held, 2000].

As a first step, we considered the following different spatio-temporal models whose fit was assessed by the Watanabe-Akaike information criterion (WAIC)(Table 1). For self-harm, based on the criterion values, we selected the model that presented the best fit (i.e. minimizing the WAIC) which had an unstructured temporal random effect, a structured temporal random effect (RW1) and type I interaction:

$$\log(\theta_{it}) = \beta_0 + b_i + \xi_t + \gamma_t(\text{RW1}) + \delta_{it}.$$

For COVID-19, the model that presented the best fit had an unstructured temporal random effect, a structured temporal random effect (RW2) and type I interaction:

$$\log(\theta_{it}) = \beta_0 + b_i + \xi_t + \gamma_t(\text{RW2}) + \delta_{it}.$$

To describe spatio-temporal patterns of both self-harm and COVID-19, we reported the mean posterior of the exponentiated θ_{it} as smoothed spatio-temporal Standardized incidence ratio (SIR).

Table 1: Different spatio-temporal models and their Watanabe-Akaike information criterion (WAIC). The minimum value of the WAIC criterion has been highlighted in bold and corresponds to the spatio-temporal model selected for self-harm and COVID-19.

	Model	WAIC	
		self-harm	COVID-19
No-interaction models	$\log(\theta_{it}) = \beta_0 + b_i + \xi_t$	267,222.39	496,922.70
	$\log(\theta_{it}) = \beta_0 + b_i + \gamma_t(\text{RW1})$	267,221.56	496,939.78
	$\log(\theta_{it}) = \beta_0 + b_i + \gamma_t(\text{RW2})$	267,221.56	497,000.56
	$\log(\theta_{it}) = \beta_0 + b_i + \xi_t + \gamma_t(\text{RW1})$	267,221.56	496,925.57
	$\log(\theta_{it}) = \beta_0 + b_i + \xi_t + \gamma_t(\text{RW2})$	267,221.62	496,928.93
Type I interaction models	$\log(\theta_{it}) = \beta_0 + b_i + \delta_{it}$	267,158.80	359,991.06
	$\log(\theta_{it}) = \beta_0 + b_i + \xi_t + \delta_{it}$	267,159,46	359,993.65
	$\log(\theta_{it}) = \beta_0 + b_i + \gamma_t(\text{RW1}) + \delta_{it}$	267,158.93	360,354.54
	$\log(\theta_{it}) = \beta_0 + b_i + \gamma_t(\text{RW2}) + \delta_{it}$	267,158.89	359,998.65
	$\log(\theta_{it}) = \beta_0 + b_i + \xi_t + \gamma_t(\text{RW1}) + \delta_{it}$	**267,158.64**	359,991.28
	$\log(\theta_{it}) = \beta_0 + b_i + \xi_t + \gamma_t(\text{RW2}) + \delta_{it}$	267,159.19	**359,967.83**

1.2 Ecological regression models

The association between between hospital admissions for self-harm and for COVID-19 was investigated using the previously selected Bayesian model, introducing each ecological variable X_{it} as a fixed-effect covariate:

$$\log(\theta_{it}) = \beta_0 + \beta_1 X_{it} + b_i + \xi_t + \gamma_t(\text{RW1}) + \delta_{it},$$

where β_1 is the log-increase of hospital admissions for self-harm incidence for 1 unit in ecological covariate. For each covariate, we reported this on natural scale ($\exp(\beta)$) as the relative risk together with its 95% Bayesian credible interval (BCI).

Initially, the covariates considered were the smoothed SIR for COVID-19 obtained in the selected spatiotemporal model in subsection 1.1. Smoothed SIRs were transformed into a qualitative variable with spatial units with SIR ≤ 1, corresponding to space-time units for which the incidence rate of hospital admissions for COVID-19 was lower than the average over the study period and area. SIR ≤ 1 served as the reference class. The other SIR classes were]1-2],]2-3],]3-4], and >4. For example, the class]2-3] corresponds to excess incidence by a factor of 2 to 3, compared with the average incidence over the whole study area and period. Given that COVID-19 might have induced a time lag in hospital admissions for self-harm, time intervals ranging from 0 to 6 months were tested.

After selection of the best model for the time lag between self-harm and COVID-19 hospital admissions, ecological covariates were added to the models through forward selection (based on a decrease in the WAIC). All the covariates were centered and reduced.

1.3 Priors

For both spatio-temporal models and ecological regressions, we specified non-informative Normal$(0, 100)$ prior distributions for $\beta_j,\ j = 0, 1$. Vague prior distributions were also specified for the inverse of the variance hyperparameters σ_l^2, $l \in \{u, v, \xi, \gamma, \delta\}$, using gamma distribution $\Gamma(1, 5.10^{-5})$.

References

[Besag et al., 1991] Besag, J., York, J., and Mollie, A. (1991). Bayesian Image-Restoration, with 2 Applications in Spatial Statistics. *Ann I Stat Math*, 43(1):1–20.

[Knorr-Held, 2000] Knorr-Held, L. (2000). Bayesian modelling of inseparable space-time variation in disease risk. *Statistics in medicine*, 19(17-18):2555–2567.
