# Supplementary material for: Association Between COVID-19 and Self-Harm: Nationwide Retrospective Ecological Spatiotemporal Study in Metropolitan France
Source: JMIR Public Health Surveill. 2024 Aug 27;10:e52759. doi: 10.2196/52759 (PMC11370185; doi:10.2196/52759)

Multimedia appendix 3

Changes in the incidence rate of hospital admissions for self-harm from January 2019 to December 2021, in people aged between 10 and 29 (yo: years old).

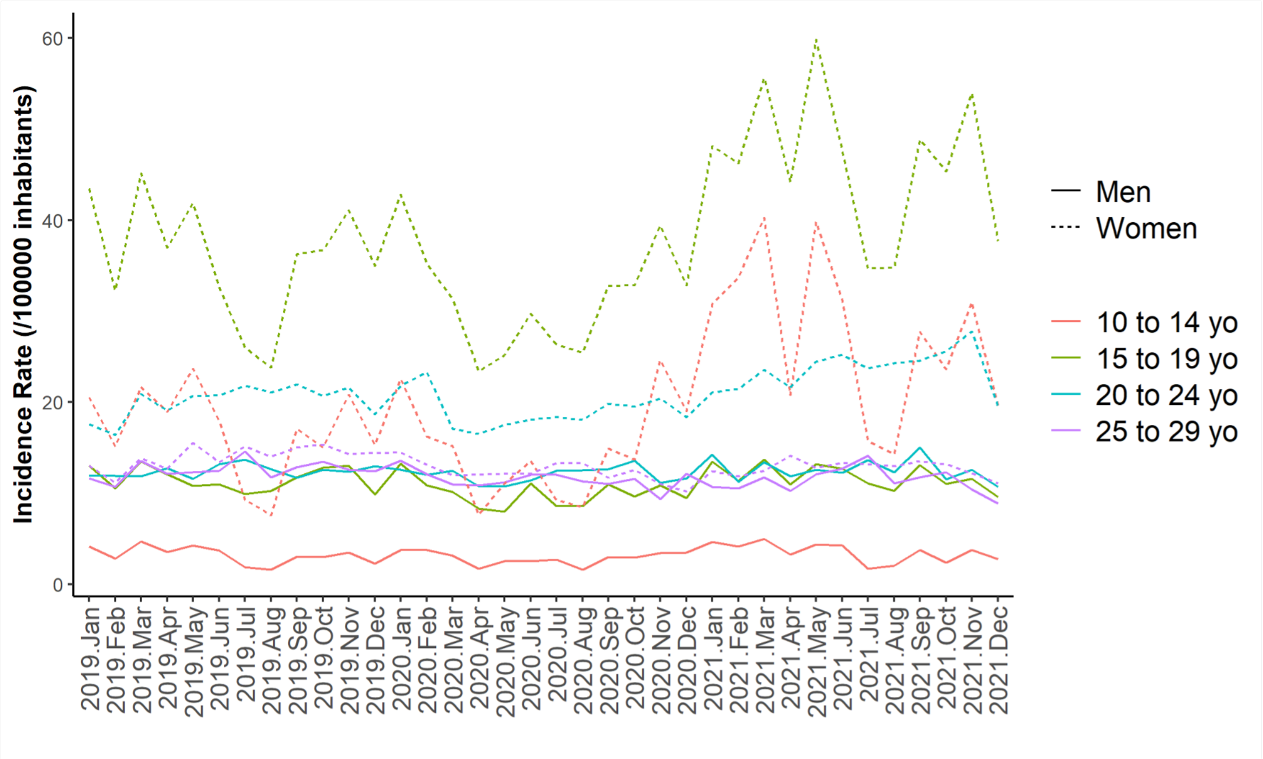

Supplement: Multimedia Appendix 3 [file publichealth-v10-e52759-s003.pdf]
